# Trephining for Intracranial Pressure

**Published:** 1891-04-04

**Authors:** 


### SURGERY.

### TREPHINING FOR INTRACRANIAL PRESSURE.

The efficacy of this treatment in cases of intracranial pressure due to abscess or tumour is fully recognised, but it is not of these we wish to speak. There are two other conditions which owe their fatality largely to this complication, but in which the same line of treatment has not, except in rare instances, been carried out; we refer to hydrocephalus and meningitis. Two questions require an answer in discussing the value of such a treatment: First, is the operation of trephining—and, if need be, exploring and draining the ventricles—safe, or so safe as to be as justifiable as, for example, an abdominal exploration? and, secondly, what results may we expect to get? With regard to the first question, we may safely affirm that merely opening the skull, *if done with rigid antiseptic precautions*, is quite devoid of danger; and as to puncturing or incising the brain or draining its cavities, the work of Horsley, Macewen, Barker, and many others goes to show that the amount of extra risk is so small as not to militate against the adoption of such a course when desirable. Last August, at the annual meeting of the British Medical Association, Mr. Mayo Robson related a case, diagnosed as a left basal meningitis, in which he trephined over the arm-centre on the supervention of right hemiplegia and aphasia, with some twitching of the arm and tonic spasm of the thumb. On opening the dura-mater the brain was non-pulsatile and compressed, and the exploring-needle, inserted in various directions, failed to find pus; finally it was directed into the lateral ventricle, and six drachms of clear fluid were drawn off. The patient made a complete recovery, with the exception of occasionally-recurring slight convulsive seizures. This operation of trephining and tapping the ventricles has been done also by Von Bergmann, Ayers, and Hersman, and Dr. Keen, who read a paper in 1888 before the College of Physicians of Philadelphia, proposing this method of treatment and formulating the technique. Lampiari reports a case of leptomeningitis in which he trephined, removing a splinter of bone. As this did not relieve the symptoms, he two days later opened the dura-mater, giving exit to a cloudy serous exudation from the arachnoid—sublimate irrigation was used—and the patient made a good recovery, aphasia and paralysis disappearing. Meiring Beck successfully trephined a case of meningitis due to middle ear disease.

A few words may be said as to the precautions that should be taken in such cases. In the foremost place undoubtedly stands strict asepsis, but this being assured, there are some other details which if strictly observed will lessen the risk of inflicting any serious damage on the brain substance, viz., avoidance of puncture in the course of the main vessels, or in parts of known importance, if possible; avoidance of lateral movements of the needle or trocar in the cerebral substance, the instrument being taken out and re-introduced for each fresh exploration; insertion of the needle in the course of the cerebral nerve fibres and vertical to the surface. For draining the ventricles, Mr. Robson recommends inserting a fine director along which Lister's sinus forceps may be passed, dilating the track and making an easy opening for the drainage tube. The ventricles may be reached by three routes—frontal, parietal, and occipital—entering the anterior or posterior cornu or the body of the ventricle; and the spot chosen can be marked out on the surface of the skull by Professor Fraser's method—as given in his "Guide to Operations on the Brain." As to the results to be looked for in these cases, these must depend largely on the particular morbid condition which is under treatment. In hydrocephalus this in its ultimate issue must depend upon the amount of injury the cerebral substance has already sustained by the slowly developing compression to which it has been subjected before operation, but as far as the operation itself is concerned, Horsley, in the exhaustive table which he drew up to illustrate his paper on "The Surgery of the Central Nervous System" at the Berlin Congress, looks for complete arrest of the hydrocephalus. He advises, however, intermittent tapping and pressure to be tried first, and if not successful drainage of the ventricles. Mr. Robson drained the lateral ventricles in a rapidly advancing hydrocephalus, but unfortunately when the brain began to shrink the drainage tube proved to be too short, slipping out of the ventricle, and so prevented proper escape of the fluid. The loss of cerebro-spinal fluid, once so much dreaded, does not seem to be in itself of much import, and so need be no bar to the performance of such operations as we have indicated.

In meningitis there are two classes to be considered. In the tubercular and so-called simple varieties the results ought to be hopeful—if we may judge by the same operation in tubercular peritonitis. The rationale of the treatment is obscure—perhaps it may consist in a readjustment of the circulation, producing an antagonistic army of phagocytes? but the fact in the one case is certain, and so the probability in the case we are considering is a strong one. The operation, therefore, is surely on the above evidence worth trying—and trying *in time*. In the septic variety of meningitis, on the other hand, our expectations will be much more gloomy—the septic element added to the effects of pressure will often baffle all our efforts. Were it possible by irrigation to remove at once all the infected lymph and pus good results might be reasonably looked for, but the mutual relations of the skull and brain forbid such a possibility in most cases. Still, we should do what we can to effect this as far as is possible, and so disinfectant irrigation should always be added to drainage in these cases, and the drainage should be as free as possible, for which purpose a large trephine opening, or more than one, is indispensable. The issue, if the patient be left alone, is certainly fatal, so no objection can be urged against any measure that promises even a remote chance of success. Above all things, it is necessary to operate early if any good is to be gained.

